# A Double-Edged Sword: The Dichotomous Roles of Dendritic Cell Subsets in Shaping CD8^+^ T Cell Immunity and Tolerance Within the Tumor Microenvironment

**DOI:** 10.3390/ijms27209064

**Published:** 2026-10-13

**Authors:** Guoyao Gao, Ke Chen

**Affiliations:** 1 Department of Urology, Tongji Hospital, Tongji Medical College, Huazhong University of Science and Technology, Wuhan 430030, China; guoyaogao224@gmail.com; 2 Hubei Institute of Urology, Wuhan 430030, China

**Keywords:** dendritic cells (DCs), CD8^+^ T lymphocytes, tumor microenvironment, immune tolerance, DC-based vaccines, antitumor immunity, autoimmunity, solid organ transplantation

## Abstract

Dendritic cells (DCs) function as a double-edged sword in tumor immunity, capable of both activating and tolerizing CD8^+^ cytotoxic T lymphocytes (CTLs). While conventional type 1 dendritic cells (cDC1s) are well-established mediators of cross-presentation for initiating anti-tumor CTL responses, existing reviews—largely overlook the tolerogenic potential of cDC1s and the context-dependent immunostimulatory capacity of cDC2s, monocyte-derived DCs (moDCs), and plasmacytoid DCs (pDCs). Furthermore, no prior synthesis has systematically reconciled the divergent functions of DC subsets across steady-state versus inflammatory conditions, migratory versus resident phenotypes, and human versus mouse model systems. The tumor microenvironment (TME) and tumor-derived exosomes further drive DCs toward an immunosuppressive phenotype, facilitating immune escape. Here, we establish a multi-dimensional framework that dissects the dichotomous roles of diverse DC subsets in regulating CTL immunity and tolerance, resolving key biological contradictions—including recent in vivo evidence that cDC2s can cross-present immune-complexed antigens via a *WDFY4*-dependent pathway shared with cDC1s—an activity that complements, but does not replace, the cDC1-restricted cross-presentation of cell-associated antigens. We also provide a hierarchical analysis of strategies to restore DC-mediated anti-tumor activity, extending the DC–CTL regulatory paradigm to autoimmunity and solid organ transplantation. A comprehensive summary table categorizes DC-based therapeutic approaches by clinical development stage. By elucidating this dynamic balancing mechanism, we offer refined insights for optimizing cancer immunotherapy design and cross-disciplinary therapeutic applications.

## 1. Introduction

Dendritic cells (DCs) occupy a critical nexus in anti-tumor immunity, uniquely positioned to orchestrate the activation, functional polarization, and ultimate fate of CD8^+^ cytotoxic T lymphocytes (CTLs) [1,2,3]. DC can also alters its functions in induction and maintenance of peripheral tolerance under some special situation [4]. This pivotal role, however, does not represent a monolithic switch for immunity; rather, it embodies a double-edged sword. On one edge, cDC1s, with their unparalleled capacity for cross-presenting tumor-associated antigens (TAAs), prime naive CTLs and unleash potent cytotoxic responses against malignant cells [5,6]. On the other edge, DCs possess an intrinsic capacity to induce profound T cell tolerance—a capability that tumors actively co-opt for immune evasion [7,8].

The TME is the master manipulator of this DC-mediated choice. It bombards DCs with a barrage of immunosuppressive signals—transforming growth factor-beta (TGF-β), interleukin-10 (IL-10), vascular endothelial growth factor (VEGF), and metabolic stressors including hypoxia and lipid accumulation—that systematically blunt their immunogenic function [9,10,11]. Rather than maturing into potent T cell activators, DCs within the TME are frequently arrested in a semi-mature or tolerogenic state. In this compromised condition, they fail to provide the essential co-stimulatory signals (Signal 2) and pro-inflammatory cytokines (Signal 3) required for robust CTL expansion and effector function. Consequently, CTLs encountering such DCs may undergo clonal anergy, exhaustion, or deletion, thereby facilitating tumor immune escape [12].

The functional landscape extends well beyond classical cDC subsets. cDC2s, pDCs, and moDCs exhibit profound plasticity, capable of either supporting or subverting CTL responses depending on the contextual cues they receive [11,13]. A 2025 study by Jo et al. showed, using mouse genetic models in vivo, that immune-complexed antigens can be cross-presented to CD8^+^ T cells by either cDC1s or cDC2s through a shared WDFY4-dependent pathway, whereas cross-presentation of cell-associated antigens remains strictly cDC1-dependent [14,15].

This finding refines—rather than abolishes—the subset-division model, and is consistent with the classic observation that CD8^+^ (cDC1-lineage), but not CD8^−^, DCs cross-prime CTLs in vivo [16]. Under inflammatory conditions, lung cDC2s can additionally acquire the Fc receptor CD64 and an IRF8-dependent maturation module, take up antigens via convalescent serum and Fc receptors, and present antigen to CD8^+^ T cells (respiratory virus model) [17].

Several excellent reviews have addressed facets of this topic. Fu & Jiang (2018) provided foundational insights into cDC1-mediated cross-priming; Hato et al. (2024) centered on specific therapeutic modalities; Tai et al. (2024) surveyed DC-based immunotherapies [5,18,19]. However, these contributions present a fragmented landscape. They seldom reconcile conflicting data arising from different physiological states (steady-state versus inflammation), cellular origins (migratory versus resident), or model systems (human versus mouse). Moreover, the discussion is typically confined to oncology, missing valuable cross-disciplinary insights from autoimmunity and transplantation biology. Recent advances—including the identification of CD207^+^ DCs as a TME-enriched state inversely correlated with CD8^+^ T cell residency and immunotherapy response [20], and the demonstration that Siglec-targeted sialylated antigens can reprogram endogenous DCs toward transplant tolerance [21]—further underscore the need for an integrated framework.

To address these gaps, this review makes three distinctive contributions. First, we establish a multi-layered analytical framework that systematically dissects the DC–CTL interaction across DC subsets, functional states, and model systems, moving decisively beyond single-subset or single-context perspectives. Second, we uniquely extend the discussion beyond oncology, examining the conserved and divergent regulatory principles of the DC–CTL axis in autoimmunity and solid organ transplantation. Third, rather than merely cataloging interventions, we provide a hierarchical analysis of therapeutic strategies—from endogenous DC modulation to engineered cell therapies—graded by mechanistic rationale and clinical evidence, including a comprehensive table categorizing DC-based approaches by development stage (preclinical, clinical trial, FDA-approved).

## 2. The Tumor Microenvironment: A Battleground for DC–CD8^+^ T Cell Interactions

The TME is a complex and dynamic ecosystem comprising not only malignant cells but also a diverse array of non-transformed stromal and immune components that collectively shape tumor progression and therapeutic response. Understanding how DCs and CD8^+^ T cells function within this milieu is a prerequisite for dissecting their dichotomous roles in tumor immunity.

### 2.1. Cellular and Molecular Composition of the TME

The TME encompasses cancer-associated fibroblasts (CAFs), endothelial cells, pericytes, and a heterogeneous immune infiltrate that includes macrophages, neutrophils, myeloid-derived suppressor cells (MDSCs), natural killer (NK) cells, B cells, CD4^+^ T helper cells, regulatory T cells (Tregs), CD8^+^ CTLs, and DCs [22]. The extracellular matrix (ECM), remodeled by CAFs and tumor cells, provides structural support while also serving as a reservoir for cytokines, chemokines, and growth factors that modulate immune cell trafficking and function. A defining feature of the TME is its profound immunosuppressive milieu. Tumor cells and stromal components secrete IL-10, TGF-β, and VEGF, which collectively impair DC maturation, promote tolerogenic phenotypes, and suppress CTL effector function [9,11]. Metabolic aberrations—including glucose deprivation, lactate accumulation, hypoxia, and altered lipid metabolism—further compromise DC and T cell functionality [23,24]. The resulting acidic, hypoxic, and nutrient-depleted microenvironment selects for tumor cell variants capable of evading immune recognition while actively disabling infiltrating immune cells.

### 2.2. Spatial Organization of DC–T Cell Interactions

Recent advances in spatial transcriptomics have revealed that DC–T cell interactions within the TME are highly organized rather than stochastic. Mulder et al. (2026), through the DC-VERSE atlas integrating single-cell RNA sequencing (scRNA-seq) data from 13 tumor types across 40 datasets, demonstrated that DCs and lymphocytes concentrate within tumor stromal regions, forming specialized immune niches [20]. Within these niches, triads of CD4^+^ T cells, CD8^+^ T cells, and DCs facilitate coordinated immune responses [25]. The spatial segregation of DC subsets within tumor nests versus stromal compartments critically determines whether productive anti-tumor immunity or immune exclusion prevails.

### 2.3. DC and CD8^+^ T Cell Positioning in Tumor Immunity

**Figure 1:**
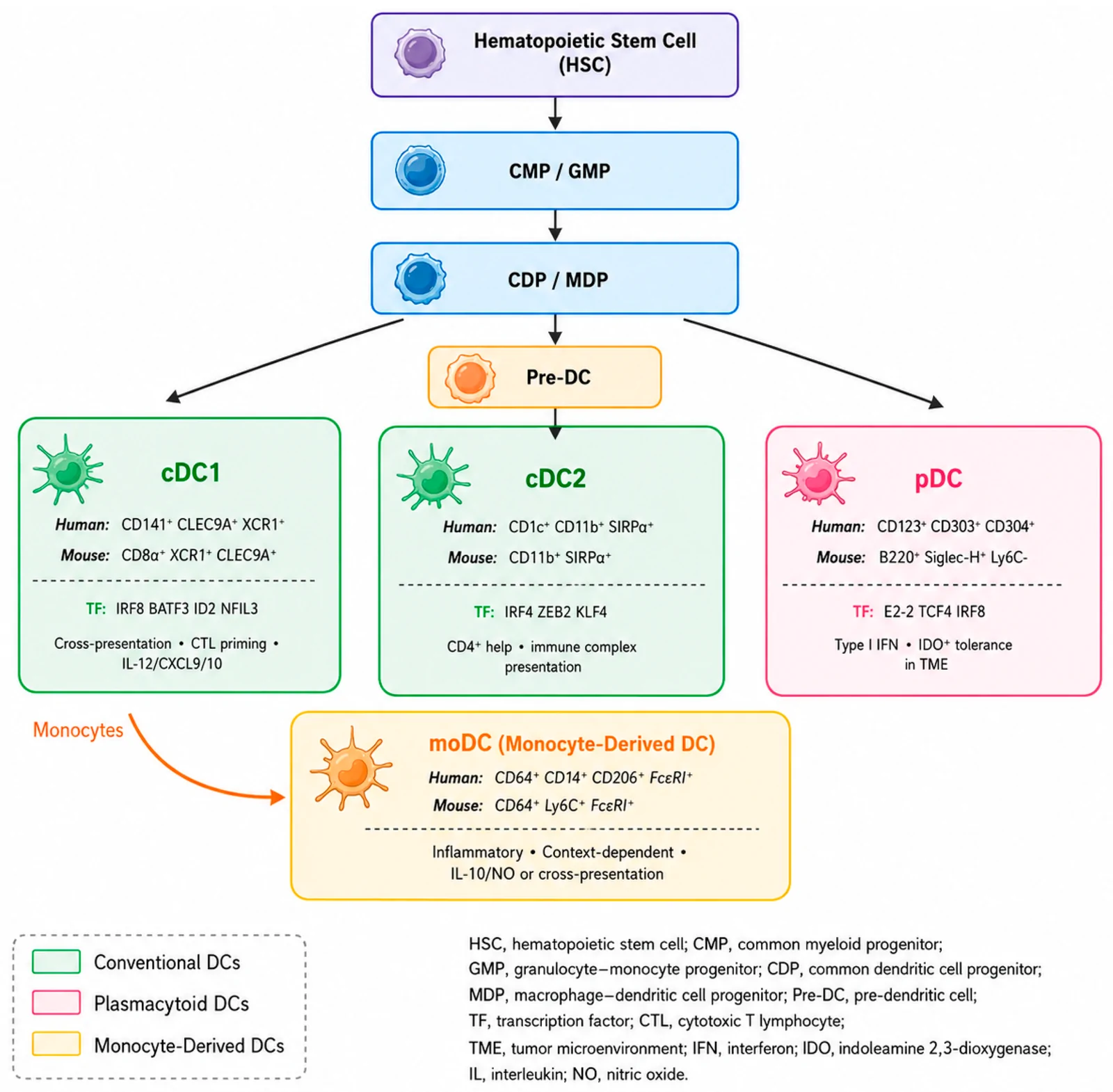
Development, Subsets, and Markers of Dendritic Cells in Human and Mouse. Schematic overview of DC ontogeny from hematopoietic stem cells (HSCs) through common DC progenitors (CDP) and pre-DC intermediates. Conventional DCs (cDC1 and cDC2), plasmacytoid DCs (pDCs), and monocyte-derived DCs (moDCs) are depicted with their defining transcription factors (TFs), surface markers, and functional specializations in both human and mouse systems.

DCs serve as the principal sentinels of the TME, continuously sampling tumor-derived antigens and migrating to tumor-draining lymph nodes (TdLNs) to initiate adaptive immune responses (Figure 1). Within tumors, cDC1s are predominantly localized in stromal regions where they recruit and activate CD8^+^ T cells through CXCL9/CXCL10 secretion [26]. The CD207^+^ DC state (associated with DC2s/DC3s), enriched within tumor nests, is inversely correlated with CD8^+^ resident memory T cell abundance, T cell clonality, and survival following immune checkpoint inhibitor (ICI) therapy [20]. CD8^+^ T cells, once recruited and activated, must overcome multiple barriers—including physical exclusion by the ECM, engagement of inhibitory receptors (programmed cell death protein 1 [PD-1], cytotoxic T-lymphocyte-associated protein 4 [CTLA-4], T-cell immunoglobulin and mucin domain-containing 3 [TIM-3]), and competition with immunosuppressive cells (Tregs, MDSCs)—to execute effective cytotoxicity [27]. The fate of the anti-tumor immune response is thus determined not solely by the presence of DCs and CD8^+^ T cells, but by their functional state, spatial proximity, and the net balance of immunostimulatory versus immunosuppressive signals within the local microenvironment.

## 3. DC Subsets in the Tumor Microenvironment

### 3.1. Conventional DCs in Tumors

Conventional DCs (cDCs), comprising the cDC1 and cDC2 subsets, represent the most critical lineage for initiating and shaping CD8^+^ T cell responses within the TME. While cDC presence broadly correlates with favorable prognosis, the ultimate impact on anti-tumor immunity is dictated by the distinct—and sometimes opposing—functions of these two subsets [2,3,28,29].

#### 3.1.1. cDC1s: Master Conductors of CD8^+^ T Cell Priming

cDC1s, identified by CD141 (BDCA-3) in humans and CD8α or CD103 in mice, are distinguished by their exceptional capacity for cross-presenting exogenous TAAs on MHC class I molecules to prime naive CD8^+^ T cells [30,31]. Their development is governed by a transcription factor network centered on interferon regulatory factor 8 (IRF8), complemented by basic leucine zipper ATF-like protein 3 (BATF3), inhibitor of DNA binding 2 (ID2), and nuclear factor interleukin 3 (NFIL3), which collaboratively program the molecular machinery for antigen cross-presentation and CTL activation [6,32]. Functionally, cDC1s perform five critical roles in anti-tumor immunity: (i) cross-presentation of TAAs via MHC I to prime naive CTLs; (ii) secretion of CXCL9 and CXCL10 to recruit effector CD8^+^ T cells into the tumor bed; (iii) production of IL-12 to promote CTL effector differentiation and memory formation; (iv) expression of C-type lectin domain family 9 member A (CLEC9A/DNGR-1) for recognition of cell death-associated antigens from necrotic tumor cells, enabling efficient antigen acquisition and cross-presentation [33]. (v) cDC1s undergo CCR7-dependent migration to TdLNs to initiate de novo T cell responses, with migratory cDC1s exhibiting superior cross-presentation compared to lymph node-resident counterparts [34]. The indispensable role of cDC1s in anti-tumor immunity has been validated across multiple model systems. Batf3−/− mice, which lack cDC1s, fail to reject immunogenic tumors and do not respond to ICI therapy [6,26]. In human cancers, cDC1 gene signatures and intratumoral abundance positively correlate with improved overall survival (OS) and response to anti-PD-1/PD-L1 therapy across melanoma, breast cancer, non-small cell lung cancer (NSCLC), and head and neck squamous cell carcinoma (HNSCC) [6].

Importantly, cDC1 function is not exclusively immunogenic, but the underlying biology differs fundamentally between the steady state and the tumor setting, and these should not be conflated. (i) Under steady-state conditions, cross-presentation of tissue/self-antigens by cDC1-lineage DCs actively induces peripheral CD8^+^ T-cell tolerance through clonal deletion and unresponsiveness, as demonstrated in vivo by DEC-205-targeted antigen delivery and tissue-antigen cross-tolerance models [35,36,37], with PD-1 and CTLA-4 acting as T-cell-intrinsic checkpoints executing this tolerance [38] (reviewed in [7]). (ii) In tumors, by contrast, the best-characterized alteration is cDC1 dysfunction—a failure to prime—rather than active tolerance induction. This distinction matters because intratumoral CD103^+^ cDC1s, although sparse, are the dominant CTL-stimulating APCs [39]. Tumor-derived PGE2 disables their capacity to orchestrate local anti-tumor CD8^+^ T-cell responses via EP2/EP4–cAMP signaling and loss of IRF8 (in vivo mouse models; the dysfunction program is conserved in human cDC1s and associated with poor patient prognosis) [8]. Likewise, PD-L1 upregulation on tumor cDC1s is embedded in a conserved maturation program (mregDC) triggered by tumor-antigen uptake and AXL signaling [40]. Neither reduced IL-12 nor elevated PD-L1, on its own, establishes active tolerization. Indeed, the best-studied active-tolerance mechanisms—IDO-driven, GCN2-mediated T-cell anergy [41] and IDO^+^ DC-driven Treg activation—were established for plasmacytoid DCs in tumor-draining lymph nodes, not for cDC1s [42], and IDO1^+^PD-L1^+^ suppressive myeloid cells in human tumors are annotated predominantly as macrophages [43]. Direct evidence that intratumoral cDC1s actively tolerize CD8^+^ T cells or drive de novo Treg expansion is therefore currently lacking.

#### 3.1.2. cDC2s: Context-Dependent Allies or Bystanders in CTL Activation

cDC2s, identified by CD1c (BDCA-1) in humans and CD11b in mice, are traditionally viewed as specialized activators of CD4^+^ T cell responses. However, emerging evidence has dramatically revised this restricted view [14,32,44]. Jo et al. (2025) demonstrated that cDC2s can physiologically cross-present immune complex antigens to CD8^+^ T cells in vivo through a WDFY4-dependent pathway shared with cDC1s [14]. Critically, while cDC1s were necessary and sufficient for cross-presenting cell-associated antigens, either cDC1s or cDC2s could cross-present immune-complexed antigens. Mice lacking cDC1s, when vaccinated with antibody–tumor antigen immune complexes, still rejected tumors via cDC2-mediated cross-priming. Huang et al. (2025) independently demonstrated that saponin-based adjuvants induce an inflammatory cDC2 (inf-cDC2) subset that becomes the dominant cross-presenting cell for CTL priming through autophagy inhibition [45], providing preliminary evidence, based on a conference abstract, that cDC2 cross-presentation capacity may be unleashed by specific inflammatory signals, though confirmatory peer-reviewed studies are warranted.

cDC2 function is profoundly influenced by the local milieu. Under homeostatic conditions, cDC2s primarily support CD4^+^ T cell responses and maintain peripheral tolerance; during inflammation, however, cDC2s upregulate co-stimulatory molecules and can directly prime CD8^+^ T cells [46]. Migratory cDC2s, which traffic from tissues to lymph nodes, carry distinct antigenic cargo compared to lymph node-resident cDC2s and can deliver tumor antigens to cDC1s in TdLNs [32]. Human cDC2s express a broader repertoire of pattern recognition receptors (TLR4, TLR5, TLR6) than mouse cDC2s [11,47], suggesting enhanced responsiveness to TME-derived danger signals. This context-dependent functional polarity—spanning steady-state versus inflammation, migratory versus resident phenotypes, human versus mouse divergence, and direct versus indirect CTL support—resolves the apparent contradiction in the literature and provides a practical framework for interpreting both existing and future studies.

Beyond the canonical cDC1/cDC2 dichotomy, single-cell analyses have resolved further heterogeneity within the human CD1c^+^ compartment, including a CD1c^+^CD163^+^CD14^+^ population first resolved by scRNA-seq of human blood and later termed DC3 [48]. DC3s were described as circulating precursors of inflammatory CD14^+^CD1c^+^CD163^+^FcεRI^+^ DCs that develop via a GM-CSF-driven pathway reported to be independent of both common DC progenitors (CDP) and monocyte-restricted progenitors (cMoP) [44]. In vitro, DC3s prime CD8^+^ T cells expressing the tissue-homing integrin CD103 via TGF-β signaling, and in luminal breast cancer, DC3 infiltration correlates positively with the abundance of CD8^+^CD103^+^CD69^+^ tissue-resident memory T cells (correlative in situ data; no clinical-outcome association was assessed) [44]. However, DC3s should not yet be regarded as an established third conventional DC subset: CD163/CD14 expression also subdivides the CD1c^+^ cDC2 compartment, within which inflammatory DC3-like cells expand in disease settings such as SLE [49], and whether DC3s constitute a bona fide lineage or a differentiation state of cDC2s or monocytes remains unresolved in ontogeny-based classification schemes [29,50].

### 3.2. Plasmacytoid DCs and Monocyte-Derived DCs

#### 3.2.1. Plasmacytoid DCs

pDCs, identified by CD123, CD303 (BDCA-2), and CD304 (BDCA-4) in humans, and CD11c^low, B220^+^, CD317^+^, SIGLEC-H^+^ in mice, are specialized type I interferon (IFN-I)-producing cells whose canonical nucleic-acid sensing is mediated by endosomal TLR7 and TLR9 signaling through MyD88–IRF7 [51]. A functional cGAS–STING pathway has also been demonstrated specifically in human pDCs, in which cytosolic DNA and cyclic dinucleotides elicit robust type I IFN responses (primary in vitro evidence) [52]. This pathway is species- and context-dependent: in a mouse malaria model in vivo, pDC-derived IFN-I production depends on TLR7–MyD88–IRF7 rather than cGAS–STING [53], and murine pDCs—unlike cDCs—fail to mount cGAS/STING-dependent IFN-I responses to cytosolic viral DNA sensing [54]; cGAS/STING in murine pDCs instead contributes to tonic IFN-I signaling that shapes pDC differentiation states [55].

Within the TME, pDCs frequently adopt an immunosuppressive phenotype: tumor-derived factors including PD-L1, hypoxia, VEGF, and TGF-beta suppress IFN-alpha production, upregulate IDO expression, and drive Treg expansion while inducing CD8^+^ T cell anergy [56,57,58,59,60]. This functional subversion is not absolute, however—context-dependent pDC subsets retain immunostimulatory capacity. For instance, OX40^+^ pDCs identified in head and neck squamous cell carcinoma synergize with cDCs to promote CD8^+^ T cell activation against tumor antigens [61]. pDCs also engage in crosstalk with NK cells and can amplify CTL efficacy in the context of oncolytic virotherapy [62,63], underscoring their potential as combination-therapy partners rather than uniformly immunosuppressive bystanders.

Therapeutically, TLR7/9 agonists represent a rational strategy to reprogram tumor-resident pDCs from a tolerogenic to an IFN-I-competent state, a concept explored further in Section 4 on DC-targeted immunotherapies.

#### 3.2.2. Monocyte-Derived DCs

**Figure 2:**
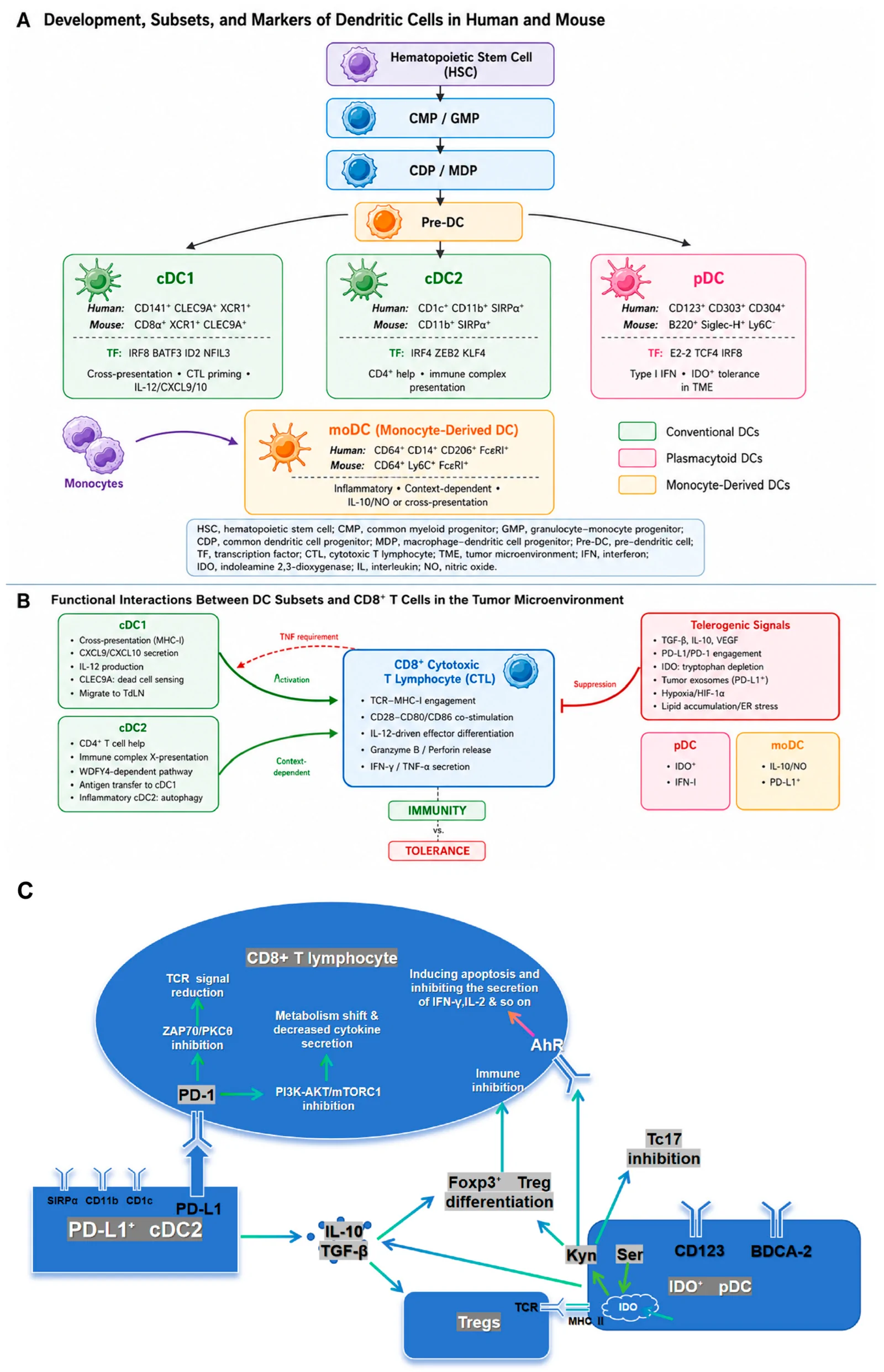
Dendritic cell subset development and functional regulation of CD8^+^ T cells in the tumor microenvironment. (**A**) Developmental trajectories of conventional DCs (cDC1, cDC2), plasmacytoid DCs (pDCs) and monocyte-derived DCs (moDCs) from hematopoietic stem and progenitor cells, with their canonical surface markers and lineage-defining transcription factors in both human and mouse. (**B**) Functional crosstalk between different DC subsets and CD8^+^ cytotoxic T lymphocytes, illustrating how immunogenic and tolerogenic signals collectively determine the balance of anti-tumor immunity and immune tolerance in the tumor microenvironment. (**C**) Molecular pathways through which PD-L1^+^ cDC2s and IDO-expressing pDCs inhibit CD8^+^ T cell activation, effector cytokine production and survival, including checkpoint ligation, metabolic interference and regulatory T cell-mediated suppression.

moDCs are defined by their monocytic ontogeny and are recruited to inflammatory sites, constituting a significant intratumoral DC population [29,64,65] (Figure 2A). A methodological caveat is warranted here: the markers routinely used to annotate moDCs—CD64 (FcγRI), CD11b, and CD206—overlap substantially with those expressed by monocyte-derived macrophages and other inflammatory mononuclear phagocytes, so that marker-based annotation alone cannot establish lineage identity. Consistent with ontogeny-based nomenclature [29], we therefore treat lineage assignment and phenotypic annotation as separate questions throughout this review. This caution is empirically grounded: in human single-cell atlases, conserved monocyte/macrophage states (e.g., IL4I1^+^PD-L1^+^IDO1^+^ macrophages) can transcriptionally resemble inflammatory moDCs [43]; in mouse tumors, Ly6Chi monocytes continuously seed tumors and renew functionally distinct macrophage subsets whose polarization tracks the local milieu [66]; and inflammatory cDC2s that acquire monocyte/macrophage features (CD64, IRF8 module) are readily confused with monocyte-derived cells [17]—all of which propagate annotation errors when lineage and phenotype are conflated.

## 4. Strategies to Restore DC-Mediated Anti-Tumor Immunity

Therapeutic strategies to re-establish DC-mediated CTL activation span a continuum from pharmacologic modulation of endogenous DCs to adoptive transfer of engineered DC products. Below, we provide a hierarchical analysis organized by mechanistic target class, with a comprehensive summary in Table 1.

Key supporting references are cited in Section 4.1, Section 4.2, Section 4.3, Section 4.4 and Section 4.5. This table encompasses both DC-directed therapies and T-cell-directed checkpoint inhibitors that act on the DC-CTL axis. Sipuleucel-T is not a purified DC product. It is an autologous leukapheresis-derived cellular product enriched for antigen-presenting cells (APCs), which are activated ex vivo with PA2024 (a recombinant PAP-GM-CSF fusion antigen). APC activation is confirmed by upregulation of CD54 and correlates with overall survival benefit in the Phase III IMPACT trial [69,70,71]. Reference [72] (Stiff et al. 2026, Cancers) reports outcomes corresponding to NCT00703105, in which autologous DCs were loaded with tumor lysate and keyhole limpet hemocyanin (KLH), rather than MUC1/WT1 peptides; the follow-up trial NCT07634094 is a distinct, more recent registration for a related Alpha-DC-1 platform combination and does not yet have an associated peer-reviewed publication. All NCT identifiers were verified against ClinicalTrials.gov records and, where available, corresponding peer-reviewed publications.

### 4.1. FLT3 Ligand and CD40 Agonism

FMS-like tyrosine kinase 3 ligand (FLT3L) is the master cytokine driving DC development from hematopoietic progenitors. Systemic administration of FLT3L expands both cDC1 and cDC2 populations and synergizes with ICIs, CD40 agonists, and radiotherapy in preclinical models [82]. CDX-301 (recombinant human FLT3L) has shown safety and pharmacodynamic activity in Phase I/II trials [82]. The REACtiVe-2 trial (2025), a Phase I study of the DC vaccine MesoPher combined with the CD40 agonist mitazalimab in metastatic pancreatic ductal adenocarcinoma (PDAC), demonstrated safety, vaccine-specific CD4^+^ T cell responses, and stable disease in 50% of patients [68].

### 4.2. DC-Based Vaccines

**Figure 3:**
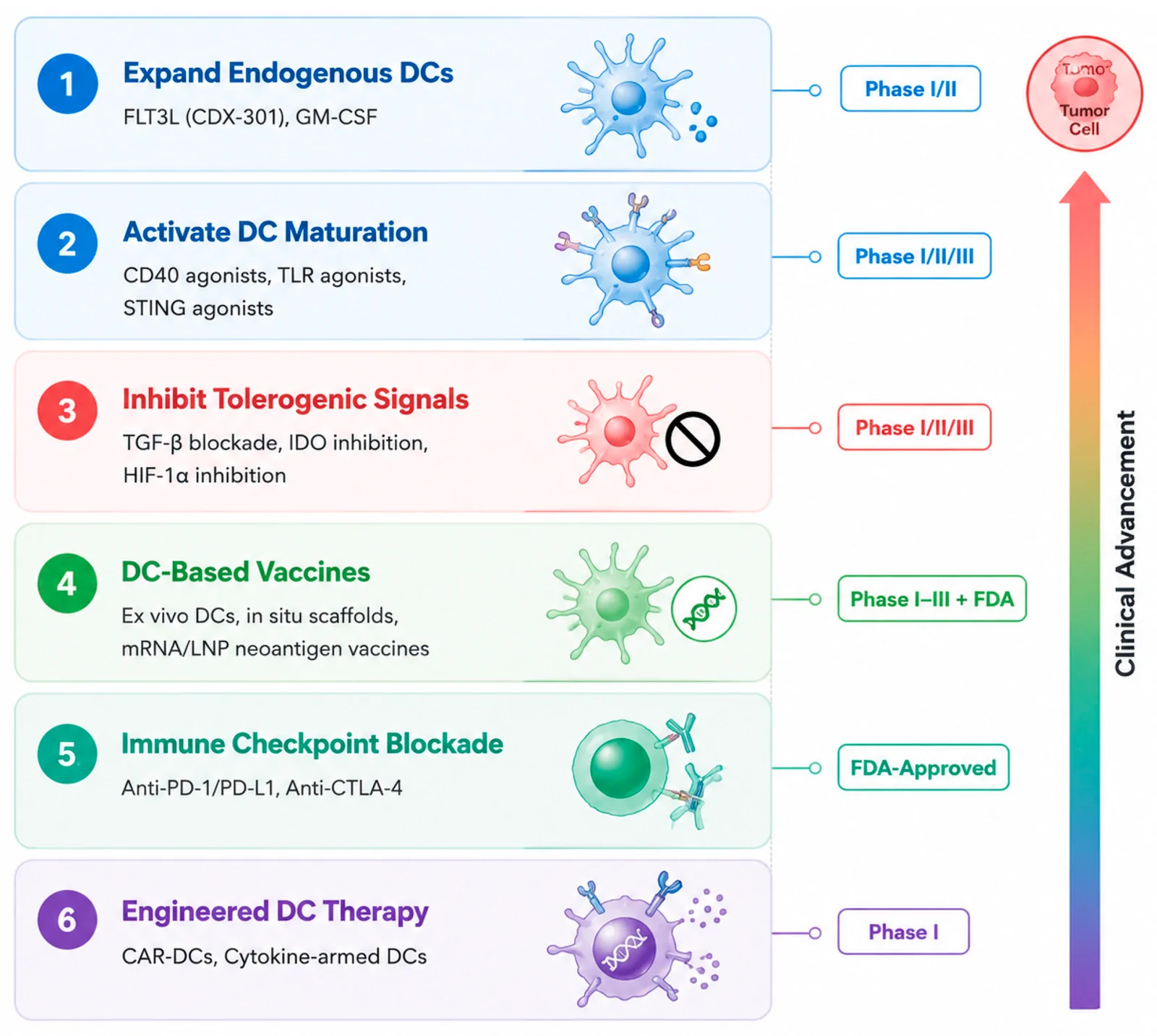
Therapeutic Strategies Targeting the DC–CTL Axis: From Endogenous Modulation to Engineered Cell Therapy. Hierarchical overview organized by target level: (**1**) expanding endogenous DCs, (**2**) activating DC maturation, (**3**) inhibiting tolerogenic reprogramming, (**4**) DC-based vaccines, (**5**) immune checkpoint blockade, and (**6**) engineered DC therapies. Clinical-stage therapies are annotated.

DC vaccines range from ex vivo-generated DCs loaded with defined antigens to in situ DC-targeting strategies. Sipuleucel-T (Provenge), an autologous product including DCs pulsed with a GM-CSF–prostatic acid phosphatase (PAP) fusion protein, remains the only FDA-approved DC-based cancer vaccine, conferring a 4.1-month median OS benefit in metastatic castration-resistant prostate cancer [69] (Figure 3). The Alpha-DC-1 pilot trial (2026) in relapsed high-grade serous ovarian cancer reported a median OS of 42.2 months and 26.3% five-year OS [72]. WDVAX, a macroporous scaffold designed to recruit and activate endogenous DCs in situ without ex vivo manipulation, demonstrated feasibility in a first-in-human Phase I trial in metastatic melanoma (2025) [73]. mRNA neoantigen vaccines encapsulated in lipid nanoparticles (LNPs) represent a rapidly advancing frontier, with Phase II trials ongoing in pancreatic cancer [75], and melanoma [74].

### 4.3. Immune Checkpoint Blockade

Immune checkpoint inhibitors (ICIs) targeting the PD-1/PD-L1 and CTLA-4 axes have transformed cancer immunotherapy. Anti-PD-1 antibodies (pembrolizumab, nivolumab) and anti-PD-L1 antibodies (atezolizumab, durvalumab) reverse CTL exhaustion by blocking inhibitory signaling in effector T cells, restoring their proliferative capacity and cytotoxic function [6,79]. Anti-CTLA-4 (ipilimumab) additionally depletes intratumoral Tregs and enhances DC licensing via CD80/CD86 engagement, promoting CTL priming. These agents are FDA-approved across more than 15 indications, including melanoma, NSCLC, renal cell carcinoma, and head and neck squamous cell carcinoma. More recently, the LAG-3 inhibitor relatlimab, in combination with nivolumab (Opdualag), received FDA approval in 2022 for unresectable or metastatic melanoma based on the RELATIVITY-047 trial, which demonstrated a median progression-free survival of 10.1 months versus 4.6 months with nivolumab alone (hazard ratio 0.75, *p* = 0.0055) [79]. This approval established LAG-3 as the third validated immune checkpoint pathway, complementing PD-1 and CTLA-4 blockade in reinvigorating exhausted CTLs.

### 4.4. TGF-Beta Neutralization and Dual PD-L1/TGF-Beta Targeting

The TGF-beta signaling axis represents a central mediator of DC suppression within the TME, inhibiting DC maturation, promoting tolerogenic phenotypes, and impairing CTL priming. Bintrafusp alfa (M7824), a first-in-class bifunctional fusion protein comprising the extracellular domain of TGF-betaRII linked to an anti-PD-L1 monoclonal antibody, was designed to simultaneously neutralize TGF-beta-mediated DC suppression and block PD-L1-mediated CTL exhaustion. Despite compelling preclinical rationale, the clinical development of bintrafusp alfa encountered successive setbacks. The Phase II INTR@PID BTC 047 trial (second-line biliary tract cancer) reported an objective response rate of only 10.1% (March 2021) [80]. The Phase II/III INTR@PID BTC 055 trial (first-line biliary tract cancer plus gemcitabine/cisplatin) was halted in August 2021 for futility, with median overall survival of 11.5 months in both arms (hazard ratio 1.23). By September 2021, Merck KGaA and GlaxoSmithKline had terminated their global co-development partnership for bintrafusp alfa, and no new trials have been initiated since [81]. While these results were disappointing, they provide instructive lessons regarding the complexity of simultaneous TGF-beta/PD-L1 targeting in unselected patient populations. Alternative TGF-beta-targeted strategies, including selective TGF-betaRI inhibitors and tumor microenvironment-restricted approaches, continue to be explored in combination with immune checkpoint blockade. The bintrafusp alfa experience underscores the translational gap between preclinical models and clinical efficacy in bifunctional immunocytokine development.

### 4.5. CAR-DCs and Engineered Dendritic Cell Therapies

Chimeric antigen receptor-engineered DCs (CAR-DCs) represent an emerging frontier that combines the antigen-targeting specificity of CAR technology with the potent T cell-priming capacity of DCs. Unlike CAR-T cells, CAR-DCs can activate endogenous polyclonal T cell responses, potentially broadening anti-tumor immunity. Preclinical studies demonstrate that CAR-DCs targeting mesothelin, HER2, and CD19 induce robust CTL activation and tumor regression in xenograft models. Phase I trials are currently evaluating safety and feasibility in solid tumors and hematologic malignancies [83].

## 5. Beyond Oncology: The DC–CTL Axis in Autoimmunity and Transplantation

The principles governing DC–CD8^+^ T cell interactions in cancer—where tolerance is undesirable and immunity is sought—have instructive parallels in autoimmunity and transplantation, where the therapeutic goal is the reverse: to restrain CTL responses and promote tolerance.

### 5.1. Autoimmune Disease

In autoimmunity, DCs that have acquired self-antigens under inflammatory conditions prime autoreactive CD8^+^ T cells, driving tissue destruction. cDC1s, through their cross-presentation capacity, are implicated in the initiation of autoimmune diabetes (type 1 diabetes), where they present pancreatic β-cell-derived antigens to autoreactive CTLs [84]. Both cDC1s and cDC2s contribute to rheumatoid arthritis, systemic lupus erythematosus, multiple sclerosis, and inflammatory bowel disease through complementary mechanisms [85]. In systemic lupus erythematosus (SLE), pDCs are central drivers of pathogenesis through TLR7/TLR9-mediated recognition of self-nucleic acid-containing immune complexes, leading to sustained type I interferon (IFN-α) production that promotes autoreactive B cell differentiation, autoantibody production, and tissue damage [86]. This pDC-IFN-α axis in SLE represents one of the most well-characterized examples of pDC-mediated autoimmune pathology and has spurred the clinical development of pDC-targeted therapies, including anti-BDCA-2 antibodies and TLR7/9 antagonists. Tolerogenic DCs engineered to present self-antigens while deficient in co-stimulation are under preclinical investigation for antigen-specific suppression of autoreactive CTLs, and nanoparticle-based delivery of antigens coupled with tolerogenic signals (rapamycin, IL-10) induces CTL anergy or deletion in model systems [87].

### 5.2. Solid Organ Transplantation

In transplantation, recipient DCs present donor alloantigens to prime donor-specific CTL responses that drive graft rejection. Kumar (2025) comprehensively reviewed regulatory DC (regDC)-mediated transplant tolerance, describing regDCs as CD11c^lo CD11b^hi CD14^+^ HLA-DR^+^ cells with high PD-L1 and FasL expression that secrete IL-10 and TGF-β, suppress donor-specific CTL proliferation, and expand Tregs [88]. A landmark 2025 study demonstrated that targeting recipient DCs with sialic acid-modified donor alloantigens via Siglec receptors prolonged skin allograft survival through a Batf3-dependent cDC1 mechanism [21]. Phase I/II trials of ex vivo-generated tolerogenic DC therapy in kidney transplantation have reported excellent safety profiles with evidence of donor-specific T cell hyporesponsiveness [88].

### 5.3. Conserved Principles Across Disease Contexts

**Figure 4:**
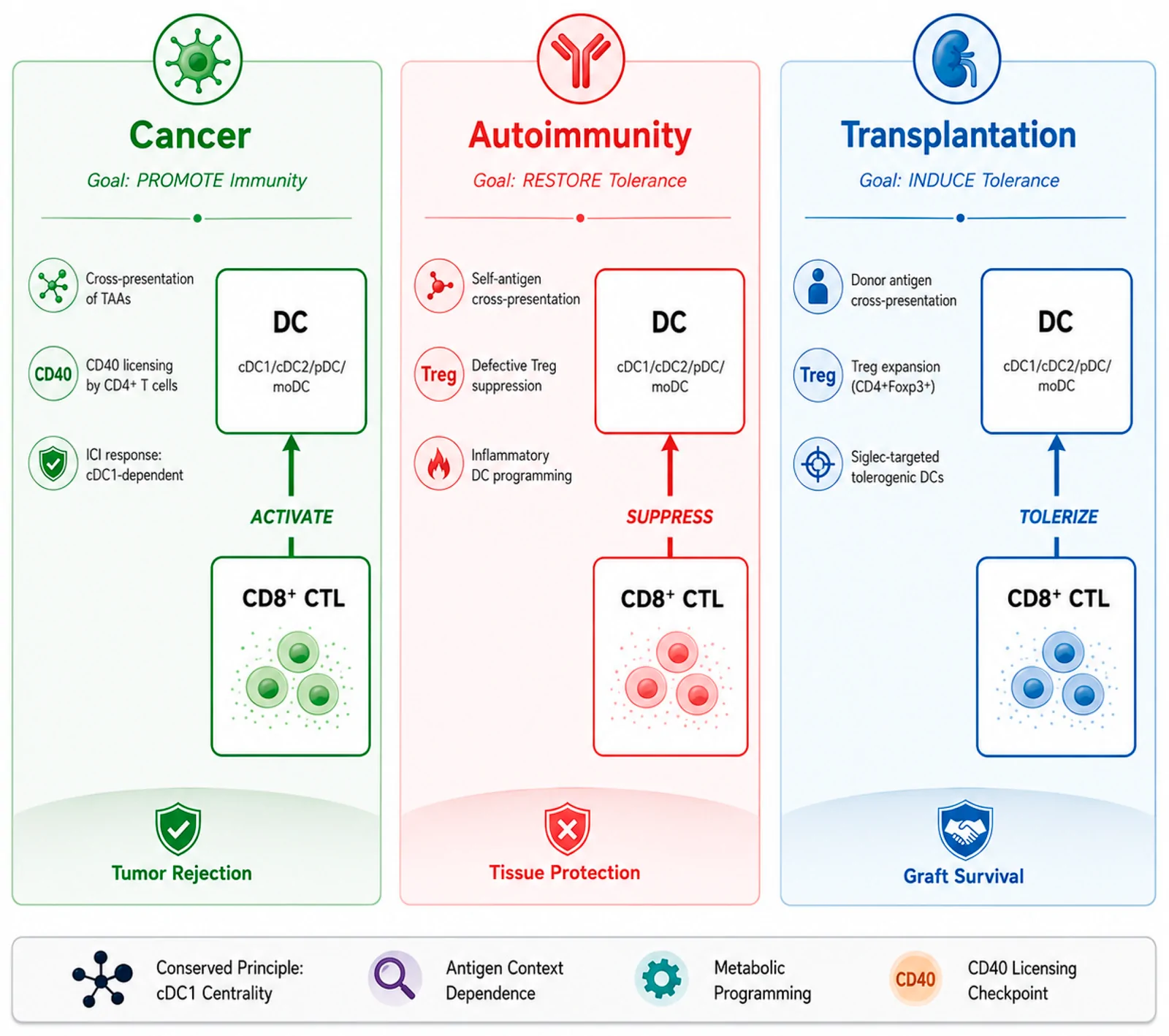
The DC–CTL Axis Across Disease Boundaries. Comparative framework illustrating conserved regulatory principles and disease-specific features of DC–CTL interactions in cancer (goal: promote immunity), autoimmunity (goal: restore tolerance), and solid organ transplantation (goal: induce donor-specific tolerance).

Comparing the DC–CTL axis across oncology, autoimmunity, and transplantation reveals three conserved principles. First, cDC1s are central conductors of CTL fate determination across all three settings. Second, the antigen format and inflammatory context in which DCs encounter antigen critically determine the immunological outcome, independent of the disease context (Figure 4). Third, metabolic programming of DCs—including the balance between glycolysis, oxidative phosphorylation, and lipid metabolism—governs their functional polarization across all disease states. These cross-disciplinary insights suggest that therapeutic strategies validated in one context may be productively repurposed across disease boundaries.

## 6. Conclusions and Future Perspectives

The DC–CD8^+^ T cell axis acts as a dynamic regulatory rheostat rather than a static molecular switch, determining the balance between anti-tumor immunity and immune tolerance. cDC1s serve as the central conductors of CTL fate determination through their unparalleled capacity for cross-presenting tumor-associated antigens, while cDC2s exhibit context-dependent functional polarity—capable of either supporting CTL activation via WDFY4-dependent cross-presentation or maintaining peripheral tolerance depending on the inflammatory milieu. moDCs and pDCs further modulate this balance through their functional plasticity in the TME. Critically, the immunosuppressive TME—through inhibitory cytokines (TGF-β, IL-10), metabolic stressors (hypoxia, lactate), and suppressive immune populations (Tregs, MDSCs)—systematically subverts DC function, skewing them toward tolerogenic phenotypes that drive CTL anergy, exhaustion, and tumor immune escape [18,89].

Comparative analysis across oncology, autoimmunity, and solid organ transplantation reveals that the DC–CTL axis operates under conserved regulatory logic across disease contexts. As detailed in Section 5.3, three principles emerge: cDC1s serve as the dominant gatekeepers of CTL fate, the antigen format and inflammatory context override subset identity in determining outcome, and metabolic programming governs DC functional polarization [23,24,90].

To accelerate clinical translation, several high-priority research directions warrant systematic investigation. First, the field must transition from single-subset analysis to multi-dimensional integration, leveraging multi-omics atlases, spatial transcriptomics, and functional immune assays to comprehensively map the DC–T cell interactome. Second, the contextual determinants governing cDC2 functional polarity—spanning tumor type, inflammatory state, anatomical location, and species—require systematic resolution to clarify persistent contradictions in the literature. Third, biomarker-driven patient stratification based on cDC1 abundance, DC functional signatures, and spatial DC–T cell organization is essential for achieving precision application of DC-modulating immunotherapies.

Beyond these priorities, several emerging frontiers hold substantial promise. DC metabolic regulation—targeting glycolysis, fatty acid oxidation, and lipid metabolism—offers novel strategies to reverse TME-induced DC dysfunction [23,90]. The combination of DC vaccination with chemotherapy or radiotherapy to induce immunogenic cell death and expose endogenous TAAs may generate synergistic anti-tumor effects [13,91,92,93,94]. Tolerogenic DC principles developed in transplantation immunology can be repurposed for autoimmune disease treatment, enabling cross-disciplinary therapeutic innovation [21,88]. Finally, advances in induced pluripotent stem cell-derived DCs (iPS-DCs), engineered to overexpress immunostimulatory cytokines such as IL-12, open new frontiers for scalable, personalized cancer immunotherapy [95].

The rapid convergence of single-cell genomics, spatial biology, synthetic biology, and materials science has created unprecedented opportunities for precise manipulation of the DC–CTL regulatory axis. Elucidating the dynamic interplay between DC subsets and CD8^+^ T cells, overcoming TME-imposed immune suppression, and optimizing iPS-DC technology, metabolic interventions, and rational combination regimens will drive fundamental immunological discoveries toward clinical impact—improving outcomes not only in oncology but across the broader spectrum of immune-mediated diseases. 

## Figures and Tables

**Table 1:** Reorganized Summary of DC–CTL Axis-Targeted and Related Therapeutic Strategies.

**A. Endogenous DC Expansion or Activation**						
**Strategy**	**Cellular Target**	**Evidence Type**	**Clinical Phase**	**Status**	**Trial ID**	**Reference**
**FLT3L (CDX-301) + anti-DEC-205-NY-ESO-1 vaccine**	Endogenous DC precursors (cDC1, cDC2, pDC expansion)	Phase II RCT (melanoma)	Phase II	Completed, published	NCT02129075	[67]
**FLT3L (CDX-301) + CDX-1140 + Poly-ICLC + RT (±pembrolizumab/tocilizumab)**	Endogenous DC precursors + CD40 agonist	Phase I (unresectable/metastatic solid tumors: melanoma, cutaneous SCC, BCC, Merkel cell carcinoma, sarcoma, HER2/neu(-) breast cancer)	Phase I	Active, not recruiting	NCT04616248	ClinicalTrials.gov registration (Study ID 1B-22-2)
**CD40 agonists (REACtiVe-2)**	Endogenous DCs + DC vaccine combo	Phase I clinical (PDAC)	Phase I (mPDAC, post-FOLFIRINOX)	Reported (n = 16, safe, 50% SD)	NCT05650918	[68]
**B. Ex Vivo DC-Based Cellular Products**						
**Strategy**	**Cellular Source**	**Evidence Type**	**Clinical Phase**	**Status**	**Trial ID**	**Reference**
**Sipuleucel-T**	Autologous leukapheresis product; APCs activated ex vivo with PA2024 (PAP-GM-CSF fusion antigen)	Phase III RCT (IMPACT) + mechanistic/correlative studies	Phase III (completed)	FDA-approved (mCRPC, 2010)	NCT00065442	[69,70,71]
**Alpha-DC-1 vaccine (advanced/low-tumor-burden high-risk ovarian carcinoma)**	Autologous DCs loaded with tumor lysate or MUC1/WT1 peptides (HLA-A2)	Pilot clinical trial, outcomes reported	Pilot trial	Completed (Loyola)	NCT00703105	[72]
**Alpha-DC-1 platform (follow-up combo)**	Same platform + IPC-CKM chemo + pembrolizumab	Protocol registration	Phase 2	Not yet recruiting	NCT07634094	ClinicalTrials.gov registration only, trial not yet initiated
**C. In Situ Antigen-Delivery or Scaffold Approaches**						
**Strategy**	**Mechanism**	**Evidence Type**	**Clinical Phase**	**Status**	**Trial ID**	**Reference**
**WDVAX scaffold**	In situ recruitment/activation of host DCs + autologous tumor lysate	Phase I, first-in-human (melanoma)	Phase I (metastatic melanoma)	Completed	NCT01753089	[73]
**mRNA/LNP neoantigen (melanoma)**	In vivo DC-targeted personalized mRNA (mRNA-4157/V940) + pembrolizumab	Phase 2b RCT (KEYNOTE-942)	Phase IIb	Active (Phase III ongoing)	NCT03897881	[74]
**mRNA/LNP neoantigen (PDAC)**	Personalized RNA neoantigen vaccine (autogene cevumeran)	Phase I clinical	Phase I	Completed	NCT04161755	[75]
**Huang et al. approach**	Saponin-based adjuvant enhancing cDC2 cross-presentation	Preclinical	Early stage	Conference abstract—awaiting peer-reviewed confirmation	N/A	[45]
**D. Non-DC-Directed Therapies Acting Downstream on the DC–T-Cell Axis**						
**Strategy**	**Molecular Target**	**Evidence Type**	**Clinical Phase**	**Status**	**Trial ID**	**Reference**
**Anti-CTLA-4 (ipilimumab)**	CTLA-4	Phase III RCT (MDX010-20)	Phase III	FDA-approved (melanoma, 2011)	NCT00094653	[76]
**Anti-PD-1 (pembrolizumab)**	PD-1	Phase III RCT (KEYNOTE-006)	Phase III	FDA-approved (melanoma + multiple indications)	NCT01866319	[77]
**Anti-PD-1 + Anti-CTLA-4 combo (nivolumab + ipilimumab)**	PD-1 + CTLA-4	Phase III RCT (CheckMate 067)	Phase III	FDA-approved (melanoma)	NCT01844505	[78]
**Anti-LAG-3 (relatlimab; Opdualag)**	LAG-3	Phase III RCT (RELATIVITY-047); PFS 10.1 vs 4.6 mo (HR 0.75)	Phase III	FDA-approved (melanoma, 2022)	NCT03470922	[79]
**Bintrafusp alfa**	TGF-β trap + anti-PD-L1	Phase II (BTC); development discontinued	Discontinued	Discontinued (2021)	Multiple	[80,81]

## Data Availability

No new data were created or analyzed in this study. Data sharing is not applicable to this article.
